# RNA Sequencing Reveals Key Metabolic Pathways Are Modified by Short-Term Whole Egg Consumption

**DOI:** 10.3389/fnut.2021.652192

**Published:** 2021-05-10

**Authors:** Amanda E. Bries, Joe L. Webb, Brooke Vogel, Claudia Carrillo, Timothy A. Day, Michael J. Kimber, Rudy J. Valentine, Matthew J. Rowling, Stephanie Clark, Kevin L. Schalinske, Elizabeth M. McNeill

**Affiliations:** ^1^Department of Food Science and Human Nutrition, Iowa State University, Ames, IA, United States; ^2^Interdepartmental Graduate Program in Nutritional Sciences, Iowa State University, Ames, IA, United States; ^3^Department of Biomedical Sciences, Iowa State University College of Veterinary Medicine, Ames, IA, United States; ^4^Department of Kinesiology, Iowa State University, Ames, IA, United States

**Keywords:** differential expression analysis, nutrigenetics/nutrigenomics, RNAseq, Sprague Dawley, miRNA–microRNA

## Abstract

Eggs are protein-rich, nutrient-dense, and contain bioactive ingredients that have been shown to modify gene expression and impact health. To understand the effects of egg consumption on tissue-specific mRNA and microRNA expression, we examined the role of whole egg consumption (20% protein, w/w) on differentially expressed genes (DEGs) between rat (*n* = 12) transcriptomes in the prefrontal cortex (PFC), liver, kidney, and visceral adipose tissue (VAT). Principal component analysis with hierarchical clustering was used to examine transcriptome profiles between dietary treatment groups. We performed Gene Ontology and Kyoto Encyclopedia of Genes and Genomes (KEGG) pathway analysis as well as genetic network and disease enrichment analysis to examine which metabolic pathways were the most predominantly altered in each tissue. Overall, our data demonstrates that whole egg consumption for 2 weeks modified the expression of 52 genes in the PFC, 22 genes in VAT, and two genes in the liver (adj *p* < 0.05). Additionally, 16 miRNAs were found to be differentially regulated in the PFC, VAT, and liver, but none survived multiple testing correction. The main pathways influenced by WE consumption were glutathione metabolism in VAT and cholesterol biosynthesis in the PFC. These data highlight key pathways that may be involved in diseases and are impacted by acute consumption of a diet containing whole eggs.

## Introduction

Eggs are a low-cost, nutrient-dense food comprised of numerous vitamins and bioactive compounds, and have been proposed to play a role in disease prevention (1, 2). Dietary whole eggs and their derived compounds (3) have been linked to several mechanisms of modulating gene expression, such as vitamin D-mediated transcriptional regulation and methyl group metabolism, by supplying choline, methionine, folate, B_12_, B_6_, and B_2_ (4). Despite the beneficial components of eggs, they remain one of the most controversial foods (5), due to their cholesterol content (6, 7). Observational studies examining the role of long-term egg intake on the risk of developing cardiovascular disease (CVD) have reported inconsistent results (8), but most recently, Dehghan and others reported no significant association between whole egg intake and major CVD events in a conglomerate of 50 studies (9). Although the role of whole egg (WE) consumption has been extensively examined in population-based studies (10, 11), relatively few studies have addressed the role of whole egg consumption in health and disease experimentally (12, 13).

Some studies have addressed the mechanistic role of individual egg components. For instance, egg yolk peptides have been shown to display anti-oxidative properties (14) and lutein, a carotenoid that is rich in egg yolk, has been demonstrated to protect dopaminergic neurons from oxidative damage in a chemically-induced mouse model of Parkinson's Disease (15). Similar effects of lutein administration have also been shown in other animal models of aging and cognitive impairment (16, 17); however, the role of these egg components and their influence on global gene expression remains not well-understood. In addition to WE decreasing oxidative stress, our laboratory has previously reported that consuming a WE-based diet reduced body weight gain in rats with type 2 diabetes (12, 13). To date, very few studies have focused on identifying the molecular mechanisms that are influenced by WE consumption in addition to the global gene expression changes in rats fed WE vs. casein (CAS)-based diets. By gaining an in-depth understanding of gene-diet interactions, we can understand how genes regulating molecular pathways are modified by short-term dietary WE consumption, allowing for future research to identify more mechanistic approaches examining the relationship between dietary WE and chronic disease.

In this study, male Sprague Dawley rats were fed WE-based diets to examine the influence of short-term WE consumption on mRNA and microRNA expression in comparison with CAS-based diets. We examined the transcriptome profiles of the prefrontal cortex (PFC), visceral adipose (VAT), liver, and kidney tissues to identify metabolic pathways that may be altered by WE consumption, and mapped these changes to microRNAs. The primary aim of this study was to initially determine if short-term WE consumption had a significant impact on gene expression in specific tissues of interest given the relevance of these tissues in disease. Furthermore, we sought to examine if changes in expression of miRNAs could be upstream of observed gene expression changes.

## Materials and Methods

The data discussed and presented in this publication have been made publicly available and deposited in NCBI's Gene Expression Omnibus (18) and can be found through GEO Series accession number GSE163193 (https://www.ncbi.nlm.nih.gov/geo/query/acc.cgi?acc=GSE163193).

### Animals and Diets

**Figure 1:**
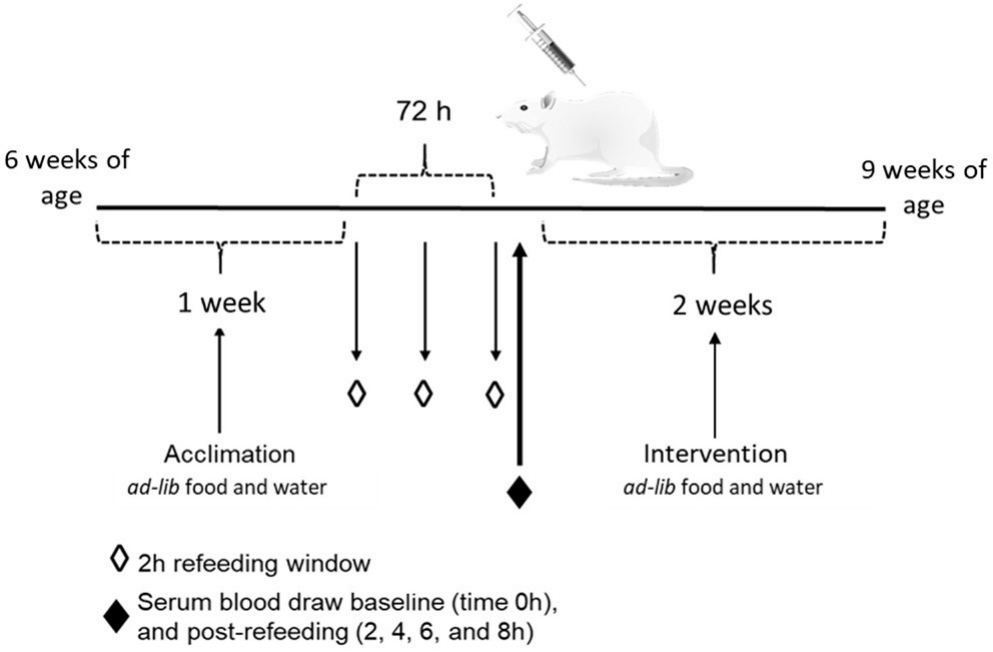
Schematic of the study design to measure the effects of whole egg consumption in a serum response curve and after a 2 week dietary intervention.

This animal study was approved by the Institutional Animal Care and Use Committee (IACUC) at Iowa State University and was performed according to the Iowa State University Laboratory Animal Resources Guidelines. Male Sprague Dawley rats (*n* = 12) were obtained at 6 week of age (151–175 g) from Charles River Laboratories (Wilmington, MA). Current studies were limited to males, building on previous studies that provide the foundation for this study. Rats were individually housed in conventional cages in a temperature-controlled environment (22 ± 2°C) using a 12-h light-dark cycle. All rats were acclimated for 1 week on a standard rat chow diet, after which they were randomly assigned to one of two dietary intervention groups. There were no significant differences in baseline body weight between the two dietary groups (*p* = 0.62). Rats were placed on either a control CAS-or WE-based diet (Research Diets, New Brunswick, NJ; Table 1) matched for 20% protein (w/w). For 72 h, animals underwent a controlled fasted-refeeding protocol to train them to consume food *ad libitum* within a 4 h time period (Figure 1) for a serum collection. After training, 7-week old animals were fasted overnight for 10 h with water provided *ad libitum* followed by controlled feeding (4 g) of either the CAS- or WE-based diet. Serum was collected *via* the tail vein at 0, 2, 4, 6, and 8 h immediately following refeeding. Following the serum time curve collection, rats were given respective dietary treatments, *ad libitum* for 2 weeks. Food intake and body weight gain were monitored daily. Following the dietary intervention, 9-week old rats underwent a 12 h overnight fast with water provided *ad libitum*; rats were anesthetized with a ketamine:xylazine cocktail (90:10 mg/kg bw) *via* a single intraperitoneal injection. Following anesthesia, whole blood was collected *via* cardiac puncture for serum sample, followed by procurement of vital organs. Euthanasia was confirmed by performing a bilateral thoracotomy. The epididymal VAT, kidney and liver, were procured, weighed, and stored in *RNA*later (Thermo Fisher, Waltham, MA). The fresh PFC was excised and rapidly micro dissected on a chilled platform. A flat edge razor was used to remove and discard the olfactory bulbs and make coronal slices through the brain. Anterior forceps of the corpus collosum (AFCC) was used as a visual landmark to identify the section from which to sub-dissect the PFC. Specifically, the darker tissue between the AFCC and the genus corpus callosum in roughly a diamond shape was dissected as the PFC. After sub-dissection the tissue was weighed and stored in *RNA*later (Thermo Fisher, Waltham, MA).

**Table 1:** Composition of the WE- and CAS-based diets fed to male Sprague Dawley rats for 2 weeks^a^.

**Ingredient (g/kg)**	**CAS**	**WE**
Casein	200	–
Whole egg^b^	–	435
Cornstarch	417	365
Glucose monohydrate	150	150
Corn oil	183	–
Mineral mix	35	35
Vitamin mix	10	10
Choline bitartrate	2	2
L-methionine	3	3
Biotin (1%)	–	0.4
Macronutrients (% total kcal)^c^		
Protein	17	17
Carbohydrate	48	48
Fat	35	35
Caloric content	4,715	4,715

a *All ingredients were purchased from Envigo except for dried whole egg (Rose Acre Farms, Guthrie Center, IA), as well as L-methionine and choline bitartrate (Sigma-Aldrich). CAS, casein-based diet, WE, whole egg-based diet*.

b *Total protein and lipid content provided by 435 g of dried whole egg was 46 (200 g) and 42% (183 g), respectively*.

c *All diets formulated to contain total protein at 20% (w/w)*.

### Large and Small RNA Extraction and Sequencing

Large- and smallRNAs were extracted using a SPLIT Total RNA Extraction Kit (Lexogen, Greenland, NH). RNA was quantified using a Qubit Fluorometer (ThermoFisher Scientific), and integrity was assessed using a Bioanalyzer 2100 (Agilent Technologies). Large RNA libraries were prepared using an automated protocol for the QuantSeq 3′ mRNA-Seq Library Prep Kit (Lexogen, Greenland, NH) and small RNA libraries were prepared using Small RNA Library Prep Kits (Lexogen, Greenland, NH). Total RNA samples were multiplexed across two lanes using an Illumina High-Seq 3000 resulting in an average of 7.5 million reads per sample prior to quality control. Small RNA libraries were also multiplexed and run on a separate lane on an Illumina High-Seq 3000 (San Diego, CA).

### Quality Control and Adapter Trimming

All large and small RNA reads were inspected using Fastqc and were trimmed to remove adapter sequences using BBDUK (BBMap: A Fast, Accurate, Splice-Aware Aligner)^1^. Read segments matching common Illumina Truseq or Nextera adapter sequences were removed for the reverse-complement or forward sequence of the adapters during processing.

### Read Alignment and Quantification

Rattus norvegicus reference genome (fasta) and genome annotation files (gtf) were obtained from the Ensembl genome browser^2^. Reads were aligned to the RNO version 6 release 94 of the Ensembl genome using the STAR v2.5.2 aligner (19). Transcripts aligning to specific genes were counted using the STAR-quantMode geneCounts function to map transcripts to each genome. Files containing microRNA counts and gene counts are located in the GEO database. Small RNA samples were processed using the smallrnaseq python tool (20), which aligns samples using Bowtie and quantifies small RNA read counts using reference fasta and gtf files from RNAcentral.org.

### Data Filtering and Quality Control

Genes were filtered out if they were not expressed in any samples or had fewer than 10 counts in half of the samples for each gene. Data filtering and alignment settings were adapted from Lexogen's QuantSeq 3′ mRNA-Seq Kit and integrated Data Analysis Pipeline on Bluebee® platform according to the manufacturer's instructions. Following quality control, analysis included 10,101 liver genes, 9,217 adipose genes, 11,607 kidney genes, and 99,36 brain genes.

### Differential Expression Analysis Using DESeq2

Read normalization was conducted using a weighted trimmed mean of the log expression ratios [trimmed mean of M values (TMM)] method to account for variable sequencing depth between samples. Differential expression analysis was conducted using the DESeq2 (21) package in the R programming language.

### Heatmaps, Principal Component Analysis, and Volcano Plots

Principal Component Analysis (PCA) was conducted for the initial clustering and characterization of RNAseq data. Hierarchical clustering was used to create a dendrogram classifying samples according to similar transcriptomic profiles using Pearson correlation coefficients. Volcano plots were constructed to visualize genes which surpass a log-fold change of >1.0 (upregulated) or <-1.0 (downregulated) to assess biological significance at adjusted *p* < 0.05. PCA and volcano plots were all constructed using MatplotLib in python version 3.2.0rc1. Heatmaps and hierarchical clustering were constructed using “heatmapper” package in R software.

### Functional Enrichment Annotations

Pathway-based analysis was performed using the Kyoto Encyclopedia of Genes and Genomes (KEGG) database that contains annotated biological functions for genes (22). All differentially expressed genes (DEGs) were additionally categorized into biological pathways using KEGG pathway analysis and Gene Ontology (GO) analysis based on cellular localization, function, and processes using the Database for Annotation, Visualization and Integrated Discovery (DAVID) v6.8 database through the online web application (DAVID Functional Annotation Bioinformatics Microarray Analysis)^3^. MicroRNA target genes were predicted through miRPath v.3 analysis tools (23). Genetic Interactome Analysis was completed on DEGs from the VAT and PFC using Cytoscape version 3.8.0 (24). STRING application—Protein Query generated a node network that allowed functional enrichment data of all DEGs to be generated. The Kyoto Encyclopedia of Genes and Genomes (KEGG) database was queried to identify the functional pathways corresponding to each gene in each species. All DEGs were cross-referenced to String—disease, specifically Diabetes, Parkinson's and Alzheimer's with a confidence interval of 0.95 and a maximum number of proteins of 500 (25).

### qRT-PCR Validation Analyses

Total RNA from each tissue was aliquoted and reverse transcribed into cDNA using the High-Capacity cDNA Reverse Transcription Kit (Applied Biosystems, Catalog # 4368813). The cDNA was diluted to 100 ng/μL and qPCR reactions were performed using 200 ng of total cDNA with primers at 300 nM concentration in 10 μL FastStart Sybr Green Master (Roche) according to the manufacturer's instructions. All qPCR reactions were conducted in a Roche LightCycler 96 Real-Time PCR System. Primers sequences for qPCR (Supplementary Table 1) were obtained from Integrated DNA Technology (IDT) and 18s RNA was used as an internal control for normalization in each tissue.

### Statistical Analysis

Data analysis was conducted in Python within an IPython notebook unless otherwise specified. Body weight and food intake group means were analyzed using an unpaired t-test to analyze the difference at *p* < 0.05 between casein and whole egg treatment groups. When applying DESeq2 for differential gene expression analysis, DESeqDataSetFromMatrix was used to generate *p*-values and adjusted *p*-values were calculated using the Benjamini-Hochberg method (26) of false discovery rate (FDR) correction. For all analyses, FDR was controlled at 1% and all adjusted *p* < 0.05 were considered significant. For functional enrichment analysis, gene identifiers were cross-validated through our differentially expressed genes on the basis of significance at FDR correction of <0.05. All qRT-PCR samples and genes were analyzed in triplicate using the Livak Delta-Delta CT method (27).

## Results

### RNA Seq Differential Expression

Differential gene expression analyses of the tissues identified two DEGs in the liver, 22 in VAT, 52 in the PFC, and none in the kidney. Of the 76 DEGs that surpassed multiple testing corrections (adjusted *p* < 0.05), one gene was differentially upregulated across both the PFC (5.6-fold increase) and VAT (3.2-fold increase) tissue: indolethylamine *N-methyltransferase* (INMT). Supplementary Table 2 describes all of the DEGs in each tissue, whereas Supplementary Table 3 contains only those with a significant *p*-value after correction in all tissues.

### KEGG and GO Functional Enrichment Analysis

To examine the functional pathways for the DEGs, mRNAs were mapped to KEGG/GO pathway terms, which are described in Supplementary Table 4. In the PFC, GO function analysis releveled genes modified by whole egg consumption were significantly enriched in Cholesterol biosynthetic pathways and lipid metabolism. Furthermore, a large genetic network of 16 genes in these two pathways were all down regulated (Supplementary Figure 1A). Of the 12 genes differentially regulated which were involved in Oxidation-reduction process, 9 were downregulated in response to whole egg consumption. In the VAT, *Gsta3, Gstz1*, and *Gstp1*, all members of the glutathione transferase activity pathway, were found to be up-regulated by whole egg consumption (Supplementary Figure 1B).

### STRING: Diseases database Enrichment Analysis

Using STRING: disease query analysis to examine disease related genes that were differentially expressed in response to WE diet, we identified one gene in VAT which is known to be associated with Diabetes. Specifically, Secreted Phosphoprotein 1 (SPP1) also known as Osteopontin was found to be down regulated in the VAT (−3.14 log2 fold change, Supplementary Table 3).

### microRNA Sequencing Differential Expression

Differential expression analyses of microRNAs identified six upregulated microRNAs and 10 downregulated microRNAs across all four tissues based on non-*adjusted p* < 0.05 (Supplementary Table 5). No microRNAs survived multiple testing correction with FDR correction at 5% value (Supplementary Table 2). miRNAs largely changed in a tissue specific manner, but two miRNAs changed in multiple tissues including miR-10b-5p which was downregulated in both the VAT and PFC (non-adjusted; *p* = 0.03 and *p* = 0.02, respectively) and miR-192-5p which was downregulated in both the liver and PFC (non-adjusted; *p* = 0.02 and *p* = 0.05, respectively).

### microRNA Gene Target Analysis

To gain a better understanding of the possible regulatory role of miRNAs, all 16 differentially expressed microRNAs (Supplementary Table 5) were mapped against their putative human genetic targets using miRPath, and cross-referenced against the DEGs in the corresponding tissue (Supplementary Table 3). Based on this mapping, three miRNAs were identified whose predicted target genes which were also differentially expressed in response to diet in the corresponding tissues (Table 2). These included miR-10b-5p which was downregulated in the PFC. Its corresponding predicted target, Arrestin Domain Containing 3 protein (ARRDC3), was upregulated. miR-192-5p was also suppressed in the PFC with nine putative targets which were upregulated. These included: SPC Membrane Recruitment Protein 1 (Amer 1), Fatty Acid Binding Protein 3 (Fabp3), Protocadherin 17 (Pcdh 17), FERM Domain Containing 4B (Frmd4b), Kinesin Family Member 1B (Kif1b), Collagen type V alpha 1 chain (Col5a1), zinc finger protein 36, C3H type-like 1 (Zfp3611), NIPA Like Domina Containing 1 (Nipal 1), and Sorting Nexin 33 (Snx33, Table 2). Additional gene targets of miR-192-5p in the PFC and miR-125b-5p in the VAT were predicted, but did not follow traditional patterns of miRNA regulation as they and the miRNAs were all down regulated (Table 2).

**Table 2:** Differentially expressed miRNAs and putative target genes in the PFC and VAT of Sprague Dawley rats fed dietary whole egg vs. casein^a^.

**MicroRNA (Log2fold change)**	**Tissue**	**Rat symbol**	**Log2fold change**	**Log fold change**	**Non-adj *p*-value**	**Human GeneID**
miR-10b-5p (−1.35)	PFC	Arrdc3	1.36E+00	1.85E+00	1.96E-05	ARRDC3
miR-192-5p (−0.82)	PFC	Amer1	1.26E+00	1.58E+00	2.81E-03	AMER1
		Blcap	−4.96E-01	−2.46E-01	1.10E-02	BLCAP
		Mylk	−4.61E-01	−2.12E-01	1.16E-02	MYLK
		Fabp3	1.33E+00	1.78E+00	1.64E-02	FABP3
		Taok1	−5.43E-01	−2.95E-01	1.74E-02	TAOK1
		Pcdh17	1.21E+00	1.46E+00	2.01E-02	PCDH17
		Frmd4b	8.81E-01	7.76E-01	2.62E-02	FRMD4B
		Kif1b	4.56E-01	2.08E-01	3.37E-02	KIF1B
		RGD1560010	−5.31E-01	−2.82E-01	3.72E-02	C4orf46
		Col5a1	1.41E+00	2.00E+00	4.17E-02	COL5A1
		Zfp36l1	4.13E-01	1.71E-01	4.86E-02	ZFP36L1
		Nipal1	6.36E-01	4.05E-01	5.19E-02	NIPAL1
		Pdhb	−4.43E-01	−1.96E-01	5.49E-02	PDHB
		Snx33	4.25E-01	1.81E-01	5.88E-02	SNX33
miR-125b-5p (−1.18)	VAT	Parm1	−1.50E+00	−2.24E+00	4.19E-06	PARM1
		Dnajc14	−9.69E-01	−9.39E-01	4.79E-04	DNAJC14

a *All target comparisons were made through mirPath*.

### Serum microRNA Refeeding Analysis

There was no significant effect of dietary treatment on serum microRNA at 0, 2, 4, 6, or 8 h immediately following refeeding based on multiple testing corrective measures using FDR adjusted *p* < 0.05 (data not shown).

### Principal Component Analysis (PCA) and Hierarchical Clustering

**Figure 2:**
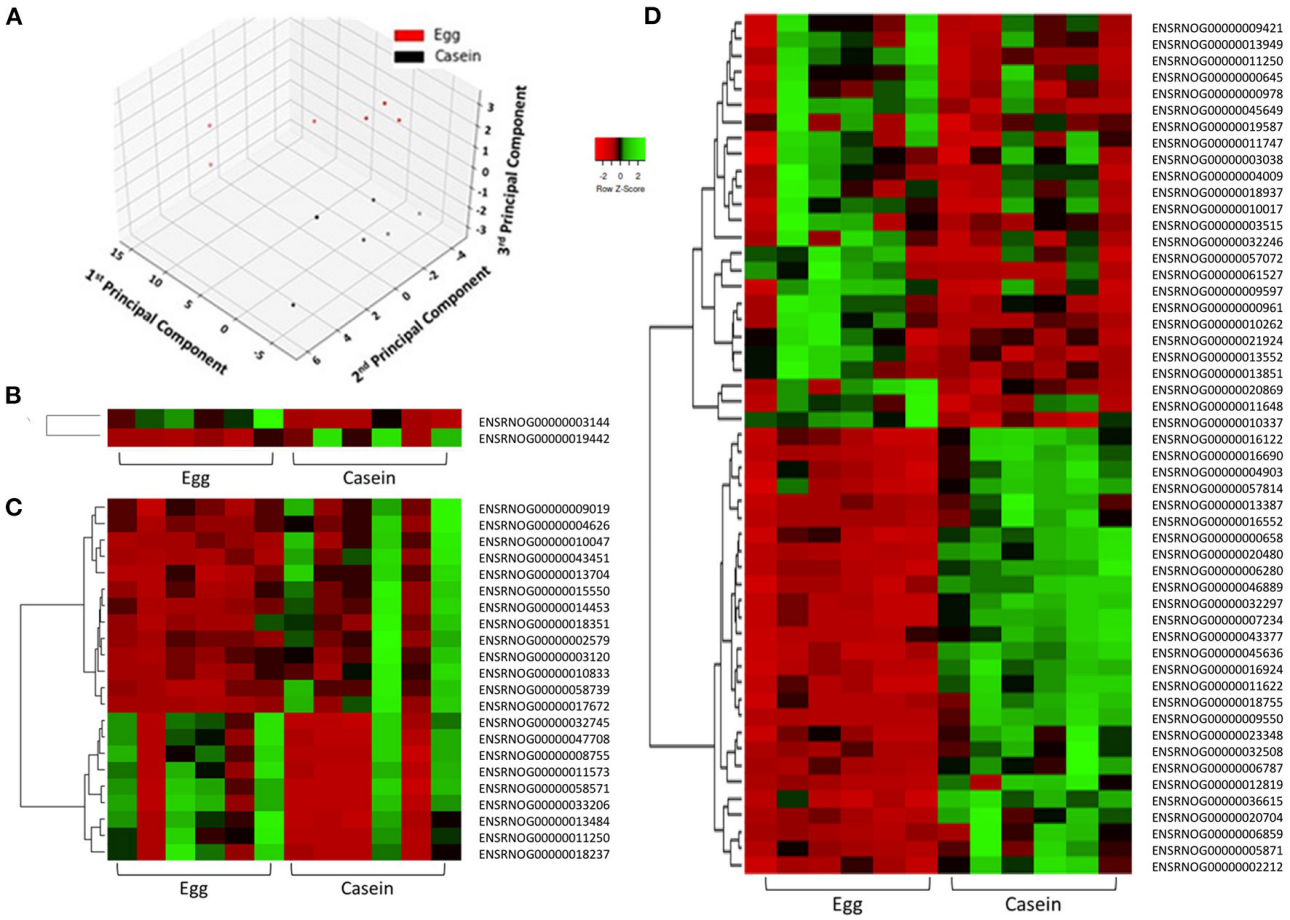
Principle component analysis demonstrates distinct separation of samples according to dietary intervention. Samples are colored in red (WE) or black (CAS, **A**). Differentially expressed genes in response to dietary intervention in: liver **(B)**, visceral adipose tissue **(C)**, and prefrontal cortex **(D)** of Sprague Dawley rats demonstrates individual and group transcriptomic response to diet using Pearson correlation coefficients (row *z*-score scale is indicated −2 Red, 0 Black, and 2 Green).

**Figure 3:**
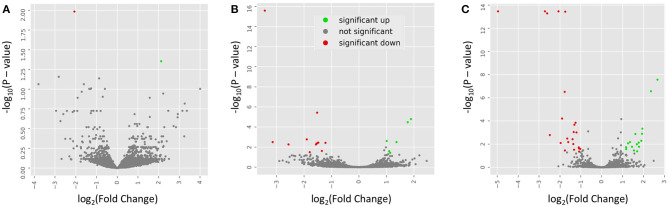
Volcano plots indicate the directionality of significant differentially expressed genes. Genes upregulated (green) or downregulated (red) by whole egg consumption, correspond to a 1.0 decrease or increase in log_2_ fold change with a log_10_ *P* > 1.0. Each panel corresponds to a tissue: **(A)** liver; **(B)** visceral adipose tissue; and **(C)** prefrontal cortex.

To examine the similarity between transcriptomic profiles, low expression genes across all tissues were filtered and data illustrated in a PCA, as shown in Figure 2A. The PCA plots indicate that these rat samples cluster by diet (WE vs. CAS). Subsequently, the top DEGs were visualized using a hierarchical clustering heatmap for each tissue that displays distinct differential expression patterns according to each diet, as shown in Figures 2B–D. To visualize the magnitude of log-fold changes, all DEGs were plotted for each corresponding tissue using volcano plots. Generally an equal distribution of both up and down regulated genes were observed in each tissue in response to WE diet. Very few genes were differentially expressed at greater than 2 log_2_ (Fold Change) in either a positive or negative direction (Figure 3A, Liver; Figure 3B, VAT; Figure 3C, PFC).

### qRT-PCR Validation

To validate the RNAseq data, five DEGs were randomly selected for qRT-PCR analysis across each tissue. The PCR gene expression validation data are contained in Supplementary Figure 2. suggesting that RNAseq and PCR results followed the same relative fold trend for each corresponding gene that was validated. A value of 1 indicates no fold change where as those values above the line indicate an increase in fold change and those below indicate a decrease.

### Food Intake and Body Weight Gain

Descriptive data indicated no significant effect of dietary treatment on food intake or total energy intake throughout the study (data not shown). Additionally, there was no statistically significant effect of dietary treatment on final body weight or cumulative body weight gain at 9 week of age after 2 weeks and 3 days on the WE diet. There were also no significant differences in organ weights following tissue collection except for the liver, where rats on the WE-based diets had 16% increased relative liver weight (*p* < 0.01).

## Discussion

Specific egg components, such as hen egg lysozymes, have been previously studied in altering gene expression in pig intestinal tissues (28), but very little information is known about how dietary WE affects endogenous gene expression across specific tissues. The beneficial role of a WE-based diet in maintaining vitamin D status and modulating adverse phenotypic outcomes in both a type 1 diabetes (T1D) and type 2 diabetes (T2D) animal models has been consistently demonstrated (12, 29); however, we have not investigated how WE consumption modulates gene expression in a standard rodent model. RNA-sequencing is a powerful tool that can elucidate the influence of dietary patterns at the level of the transcriptome. It is important to examine the global effects of WE consumption in a healthy rodent model to better understand the pathways that link WE and chronic diseases like type 2 diabetes. By assessing the genes that are influenced by WE, we can contribute to scientifically sound intervention strategies using dietary WE as a means of disease mitigation. In this study, next generation sequencing revealed that consuming WE for 2-weeks significantly modified the expression of 76 different genes across the PFC, liver, and VAT.

In the PFC, where the largest number of genes were changed, the top three enriched GO pathways were cholesterol biosynthetic process, fatty acid biosynthetic process and oxidation-reduction process. A genetic network of 16 genes highly represented in the cholesterol biosynthetic process and lipid metabolism were all down regulated. In addition, we found a large number of genes (9/12) in the oxidation-reduction process pathway to be downregulated. Dysregulation of oxidation-reduction pathways is a common thread in dementia, and modulation of this pathway is thought to be important in brain health (30). The observed reduction in expression of oxidation-reduction pathway genes in our study could be due to the high choline content of WE. Recently, associations have been observed between dietary choline intake and reduced risk of incident dementia and improved cognitive performance (31). One gene found to be highly down regulated in the oxidation-reduction pathway was Squalene epoxidase (Sqle), a rate-limiting gene in the sterol biosynthesis pathway (32), which was significantly downregulated (−31-fold) within the PFC of rats fed WE-based vs. CAS-based diets. Sqle is important for steroidal synthesis, and previous studies have demonstrated that ablation of Sqle may disrupt tumorigenesis, owing to blunted cholesterol biosynthesis (33). Moreover, the dysregulation of Sqle has been observed during the onset of diabetes (34, 35). Ge et al. (36) indicated a significant upregulation in the expression of Sqle, as well as an abundance in the protein in a chemically-induced diabetic animal model (37). Interestingly Ding et al. (35), conducted a cross-sectional study in an obese population of men, examining the relationship between body mass index and DEGs. They reported the close association between body mass index and increased expression of Fads1, Sqle, Scd, Cyp51a1, whereas in a weight-loss intervention study, Sqle expression was significantly reduced. Similar to these findings, we observed downregulation of Fads1 and Sqle in animals fed the WE- as compared to the CAS-based diets. A small number of genes included in the oxidation-reduction pathway were upregulated including two cytochrome P450 genes, Cyp2c22, and Cyp4A1 in the PFC of the animals fed WE- vs. CAS-based diets. While additional research is needed, CYPS are known to play a role in susceptibility to neurotoxins, and the risk of developing certain CNS diseases (38).

In the VAT, functional annotation of the DEGs to GO terms indicated a significant enrichment of genes involved in the glutathione S-transferase (GST) activity pathway including glutathione S-transferase zeta 1 (Gstz1), glutathione S-transferase A3 (Gsta3), and glutathione S-transferase pi 1 (Gstp1), which were upregulated 1.9-, 2.6-, and 3.7-fold, respectively, in the VAT of the Sprague Dawley animals fed WE-based diets. Previously, it was reported that deficiency of glutathione-related pathways alters antioxidant responses (39), suggesting that our data may indicate an upregulation of the catalytic GST enzymes, which function in glutathione conjugation for detoxification reactions, with WE consumption providing protection against oxidative stress. In a recent study, a Gstp1 polymorphism was associated with increased glucose intolerance and greater androgen production in non-obese women with polycystic ovary syndrome (40). Moreover, the literature indicates that dysregulated GST production has been implicated in conditions of obesity and T2D (41). It was previously reported that glutathione metabolism is regulated by egg yolk peptide consumption in a porcine model of oxidative stress (42), while in Zucker Diabetic Fatty rats WE consumption led to an upregulation in 14 GST-related enzymes across numerous tissues (43). In a meta-analysis of clinical studies with long-term egg consumption, there were no observed effects of WE consumption on blood inflammatory markers (44). Other researchers have reported elevations in endothelial and arterial inflammation from WE consumption (45) and some report vascular inflammation to be exclusive to egg white consumption and not WE (46, 47). It is important that we examined these differences in gene expression, as we identified upregulation not only in VAT, but also observed an increase of glutathione S-transferase mu 2 (Gstm2) expression in the PFC of rats fed WE-based diet. These are important considerations, as the data from clinical trials regarding WE consumption on inflammation-mediated cardiovascular disease are nuanced and remain contradictory (7, 48–50). When specifically examining genes associated with disease we identified SPP1 which was down regulated 8.8-fold in VAT after consumption of the WE diet. SPP1 also known as Osteopontin is an inflammatory cytokine which is highly upregulated in obesity and has been shown to be a key player for local adipose tissue macrophage proliferation which underlies insulin resistance and type 2 diabetes (51). Importantly Osteopontin has been found to be required for the early onset of high fat diet-induced insulin resistance in mice (52). The down regulation of this gene may account for some of the benefits observed in longer term consumption of WE diet in the Zucker Diabetic Fatty Rats (12, 13) and requires further mechanistic study.

To better understand what may be driving the changes in gene expression in response to WE diet, we performed small RNA sequencing to examine miRNAs. Previous research by Zempleni et al. has reported robust findings for meaningful dietary microRNA absorption from milk and egg sources. They report a postparandial increase in plasma miRNAs in humans suggesting biological value of dietary-derived microRNAs on downstream target genes (53, 54). While it is controversial whether dietary consumption directly influences circulating microRNA status (55), using a more highly processed whole egg product, we did not observe any serum changes in circulating microRNAs between the two dietary treatments in the 8 h post meal consumption. However, we hypothesized that over a longer period of diet consumption, the tissue microRNA profile may change due to diet; thus, we examined the microRNA expression changes in rats following a 2-week consumption period.

Six upregulated and ten down regulated miRNAs were identified across all tissues profiled. However, when the miRNAs putative gene targets were cross-referenced against the differentially expressed genes within each respective tissue, only three microRNAs had putative targets which were also changed in expression. Of these three, two were identified in the PFC (miR-10b-5p and miR192-5p) and one was in the VAT (miR-125b-5p) all three miRNAs were down regulated. ARRDC3, a target gene for miR-10b-5p exhibited a ~1.9-fold change as a result of WE consumption with a non-adjusted *p*-value of (*p* = 0.027) was the most relevant to observed phenotypes in our work with the Zucker Diabetic fatty rats. The biological significance of ARRDC3 has been examined in both human and animal studies (56, 57). It has been suggested that ARRDC3 is critically involved in obesity and energy expenditure, owing to its function in fat pad development and role in thermoregulation (58). Although there are no mechanistic studies to date examining the direct role of miR-10b-5p on ARRDC3 as it relates to obesity or subsequent diseases, to date, one study explored the association between miR-10b-5p in children with obesity (59). In a population of normal weight and overweight or obese adolescents, they assessed circulating microRNAs and identified that miR-10b-5p is an obesity-associated microRNA, as it highly correlated with body mass index *z*-scores (59). Based on our consistent results of WE consumption on reduced weight gain during type 2 diabetes, the results of this study could provide insight into the mechanisms involved in weight reduction. Future mechanistic studies exploring the relationship between miR-10b-5p, ARRDC3 as it relates to obesity, and type 2 diabetes are warranted.

There are several strengths and limitations to consider in our research study. Given the limited data on the mechanisms related to whole egg consumption and disease, our profiling experiments provide valuable data on the altering effects of whole egg consumption on gene expression. Data of this magnitude will help better define target pathways and genes that may be used to further examine the influence of nutrition on disease progression. Limitations such as the duration of dietary intervention should be noted. Furthermore, our functional pathways analysis does have limitations. The first being that pathway analysis does not account for the tissue- and cell-specific function of genes in a given pathway. Additionally, not all gene variants are represented in pathway analysis tools, limiting the scope of tissue specificity in related pathway changes.

### Conclusion

In this study, we examined the effects of consuming a WE-based diet for 2 weeks on gene expression across multiple tissues in healthy rats. Our main results revealed 76 novel DEGs across the PFC, liver, VAT, and kidney transcriptomes. For VAT, GO analyses highlighted that glutathione metabolism was upregulated by WE consumption. Furthermore, SPP1 was identified to be highly down regulated in the VAT after WE consumption and should be further explored as it relates to outcomes of WE consumption and Diabetes. In the PFC, a large network of genes from the cholesterol biosynthetic and lipid metabolic process were all down regulated in the PFC. In addition, multiple genes involved in the oxidation-reduction process in the PFC were also altered by WE consumption. These results indicate that, even short-term, WE-based diets modulates the expression of genes. Together, these results may provide information for follow up in models where these genes and pathways are involved in the pathogenesis of disease.

## Data Availability Statement

The datasets presented in this study can be found in online repositories. The names of the repository/repositories and accession number(s) can be found at: https://www.ncbi.nlm.nih.gov/geo/query/acc.cgi?acc$=$GSE163193.

## Ethics Statement

The animal study was reviewed and approved by Institutional Animal Care and Use Committee (IACUC) at Iowa State University.

## Author Contributions

AB, JW, TD, MK, MR, SC, KS, and EM conceptualized the original research. AB, JW, and EM curated and analyzed the data. Methodology of the research studies was developed by KS, EM, AB, and JW. Investigation, experiments, and animal handling was performed by AB, JW, BV, and CC. The original draft of the manuscript was written and prepared by AB and JW. Funding acquisition, project administration and writing, reviewing, and editing was carried out by AB, JW, TD, MK, MR, SC, KS, and EM. All authors contributed to the article and approved the submitted version.

## Conflict of Interest

The authors declare that the research was conducted in the absence of any commercial or financial relationships that could be construed as a potential conflict of interest.

## Footnotes

^1^*BBMap: A Fast, Accurate, Splice-Aware Aligner*. Available online at: https://escholarship.org/uc/item/1h3515gn (accessed January 28, 2020).

^2^*Ensembl genome browser 99*. Available online at: https://useast.ensembl.org/index.html (accessed March 25, 2020).

^3^*DAVID Functional Annotation Bioinformatics Microarray Analysis*. Available online at: https://david.ncifcrf.gov/home.jsp (accessed March 23, 2020).

## Supplementary Material

Supplementary Figure 1 Network of interactome representing STRING Network interactions of DEGs in specific tissues. Prefrontal Cortex **(A)** GO indicated enrichment of involved network genes in cholesterol biosynthetic process (FDR 4.96E^−16^) and lipid metabolic process (FDR 4.09E^−13^) and Visceral Adipose Tissue **(B)** GO indicated enrichment of involved network in Glutathione Transferase activity (FDR 5.1 E^−04^). Networks connected through “edge” lines with thickness represented by STRING confidence score. Colors in nodes represent gene expression changes, upregulated orange and downregulated blue. Red border indicates gene is identified in STRING network as a Diabetes associated gene.

Supplementary Figure 2 Fold change comparisons between qPCR and mRNA sequencing of several genes validating the directionality of sequencing results. Those above 1 indicate a fold increase in expression due to diet. Those below 1 indicated a decrease fold expression in relation to diet. Fold changes are significant at *p* < 0.05 unless otherwise indicated. Blue bars represent qPCR data and orange bars represent RNAseq data. Each tissue is indicated by different shading with the name of the tissue at the top. PFC, Prefrontal Cortex; VAT, Visceral Adipose Tissue; ns, not significant.

Supplementary Table 1 Primer sequence as used for qPCR.

Supplementary Table 2 Raw differentially expressed genes in the PFC, liver, adipose, and kidney.

Supplementary Table 3 List of significantly differentially expressed genes in the PFC, liver, adipose, and kidney.

Supplementary Table 4 Kegg pathway and gene ontology (GO) analysis.^a,b^.

Supplementary Table 5 Differentially expressed microRNAs between Sprague Dawley rats fed dietary whole egg compared to casein control.

## Nomenclature

Akrc1, aldo-keto reductase family

CAS, casein

Cyp51, Lanosterol 14α demethylase

DAVID, Database for Annotation, Visualization and Integrated Discovery

DEGs, differentially expressed genes

DMT, N,N-dimethyltryptamine

FDR, false discovery rate

GO, gene ontology

GST, glutathione S-transferase

GSTP1, glutathione S-transferase pi 1

GSTZ1, glutathione S-transferase zeta 1

INMT, indolethylamine N-methyltransferase

KEGG, Kyoto Encyclopedia of Genes and Genomes

KNN, K-nearest-neighbor

PCA, Principal Component Analysis

PFC, prefrontal cortex

RIN, RNA integrity number

SAM, S-Adenosyl-methionine

Sqle, Squalene epoxidase

T1D, type 1 diabetes

T2D, type 2 diabetes

TMM, trimmed mean of M values

VAT, visceral adipose tissue

WE, whole egg
