# Supplementary figures and images for: RNA Sequencing Reveals Key Metabolic Pathways Are Modified by Short-Term Whole Egg Consumption
Source: Front Nutr. 2021 May 10;8:652192. doi: 10.3389/fnut.2021.652192 (PMC8141817; doi:10.3389/fnut.2021.652192)

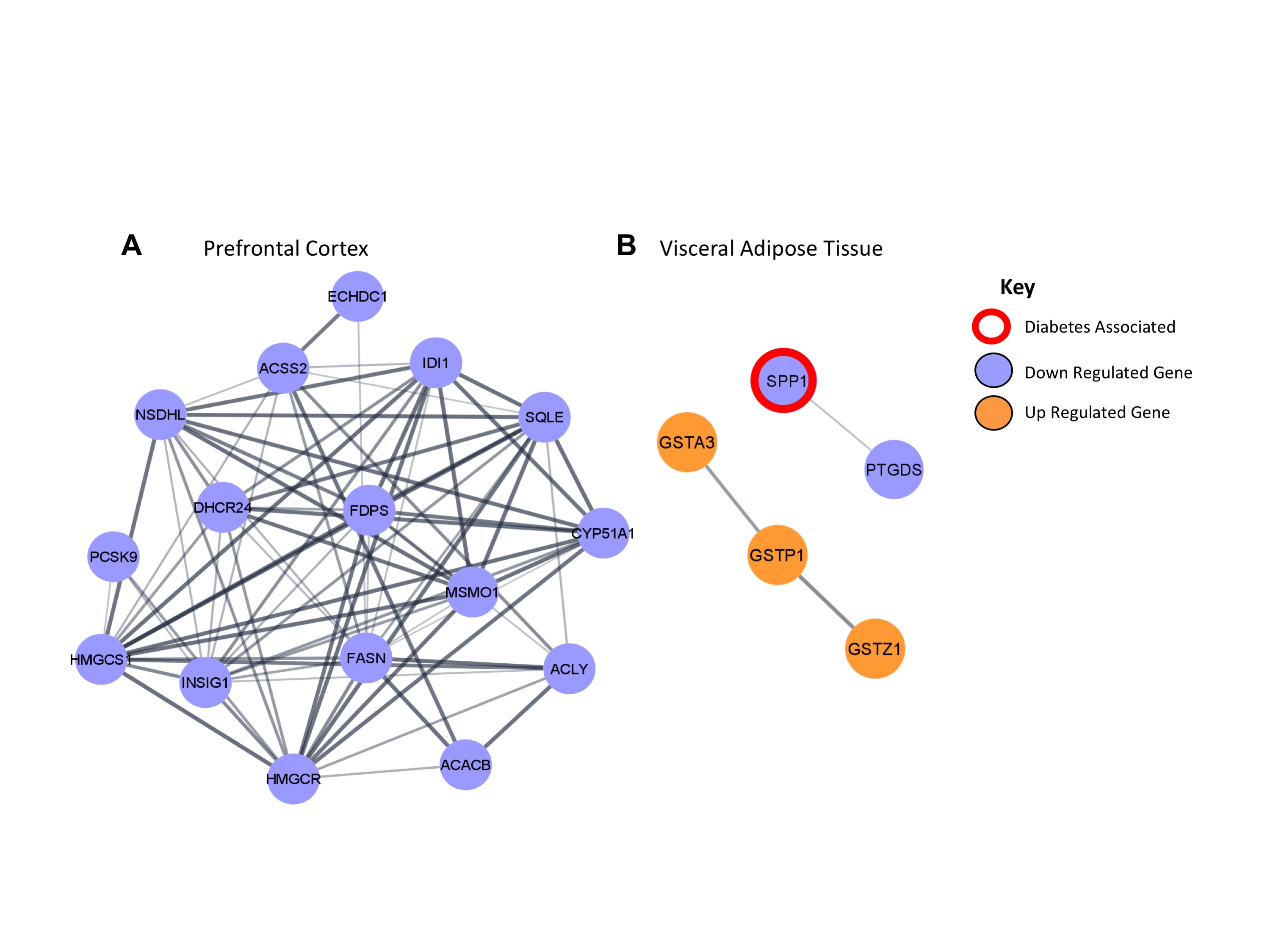

Supplement: Supplementary Figure 1 — Network of interactome representing STRING Network interactions of DEGs in specific tissues. Prefrontal Cortex (A) GO indicated enrichment of involved network genes in cholesterol biosynthetic process (FDR 4.96E−16) and lipid metabolic process (FDR 4.09E−13) and Visceral Adipose Tissue (B) GO indicated enrichment of involved network in Glutathione Transferase activity (FDR 5.1 E−04). Networks connected through “edge” lines with thickness represented by STRING confidence score. Colors in nodes represent gene expression changes, upregulated orange and downregulated blue. Red border indicates gene is identified in STRING network as a Diabetes associated gene. [file Image_1.jpeg]

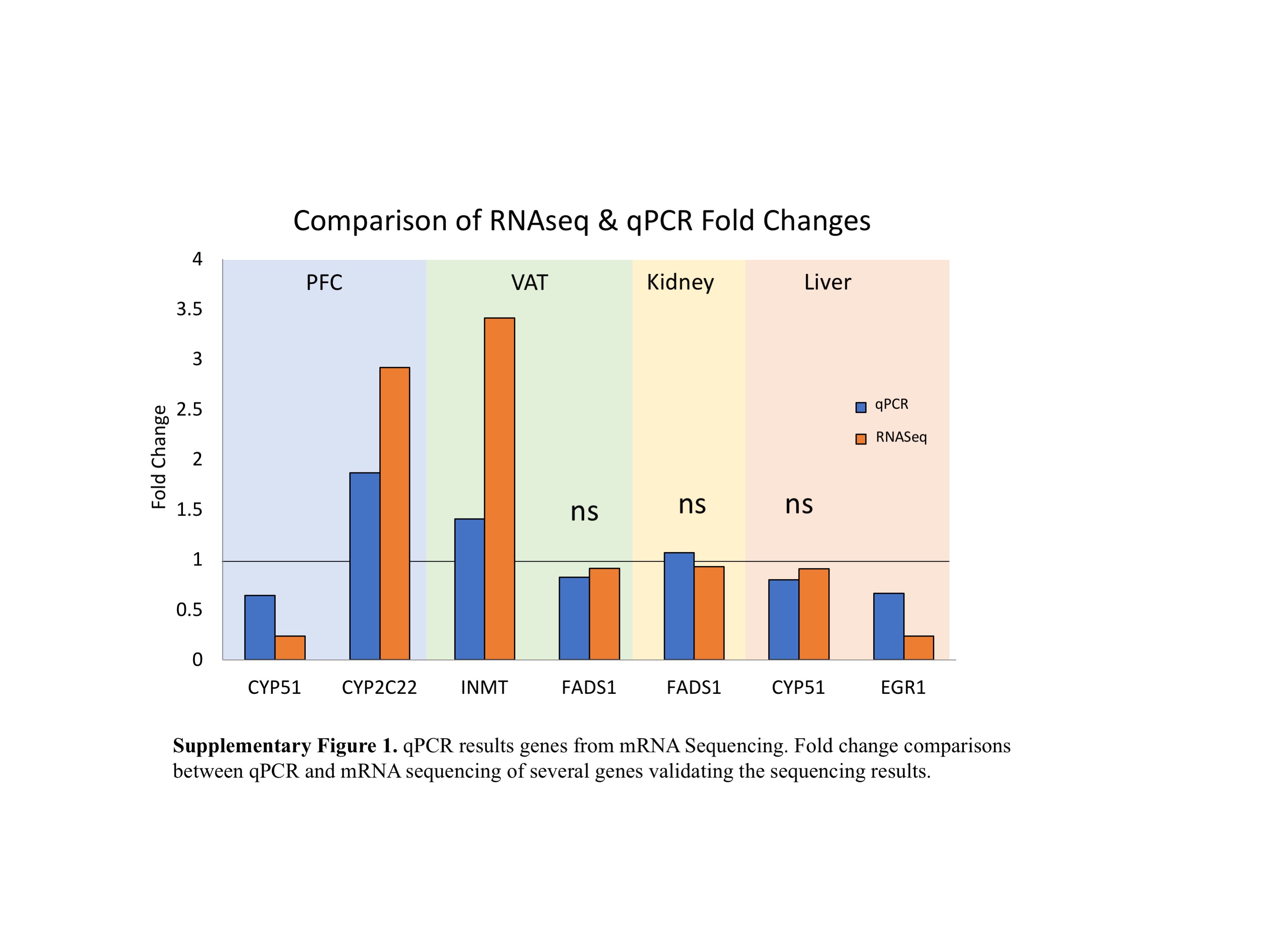

Supplement: Supplementary Figure 2 — Fold change comparisons between qPCR and mRNA sequencing of several genes validating the directionality of sequencing results. Those above 1 indicate a fold increase in expression due to diet. Those below 1 indicated a decrease fold expression in relation to diet. Fold changes are significant at p < 0.05 unless otherwise indicated. Blue bars represent qPCR data and orange bars represent RNAseq data. Each tissue is indicated by different shading with the name of the tissue at the top. PFC, Prefrontal Cortex; VAT, Visceral Adipose Tissue; ns, not significant. [file Image_2.jpeg]
