# Supplementary material for: RNA Sequencing Reveals Key Metabolic Pathways Are Modified by Short-Term Whole Egg Consumption
Source: Front Nutr. 2021 May 10;8:652192. doi: 10.3389/fnut.2021.652192 (PMC8141817; doi:10.3389/fnut.2021.652192)
Supplement: Supplementary Table 3 — List of significantly differentially expressed genes in the PFC, liver, adipose, and kidney. [file Table_3.docx]

**Supplementary Table 3.** List of Significantly Differentially Expressed Genes in the PFC, Liver, and Adipose^1^.

| **Tissue** | **Ensembl_ID (ENSRNO)** | **Symbol** | **Gene Name** | **L2FC** | **P-value^3^** |
| --- | --- | --- | --- | --- | --- |
| **PFC Up-regulated** | G00000010262 | Hdc | histidine decarboxylase | 2.67 | 2.77E-08 |
|  | G00000011250 | Inmt | indolethylamine N-methyltransferase | 2.36 | 2.76E-07 |
|  | G00000000961 | Glt1d1 | glycosyltransferase 1 domain containing 1 | 1.95 | 4.70E-04 |
|  | G00000061527 | Gck | glucokinase | 1.94 | 1.29E-03 |
|  | G00000013851 | Spry4 | sprouty RTK signaling antagonist 4 | 1.90 | 5.19E-03 |
|  | G00000010337 | Slc13a2 | solute carrier family 13 member 2 | 1.79 | 1.70E-02 |
|  | G00000013552 | Scd | stearoyl-CoA desaturase | 1.78 | 7.50E-03 |
|  | G00000020869 | mrpl9 | mitochondrial ribosomal protein L9 | 1.70 | 4.23E-02 |
|  | G00000032246 | Acsm3 | acyl-CoA synthetase medium-chain family member 3 | 1.69 | 1.03E-02 |
|  | G00000009597 | Cyp4a1 | cytochrome P450, family 4, subfamily a, polypeptide 1 | 1.62 | 1.34E-03 |
|  | G00000021924 | Cyp2c22 | cytochrome P450, family 2, subfamily c, polypeptide 22 | 1.55 | 3.56E-02 |
|  | G00000057072 | Slc12a3 | solute carrier family 12 member 3 | 1.46 | 1.70E-02 |
|  | G00000045649 | Arrdc3 | arrestin domain containing 3 | 1.36 | 6.69E-03 |
|  | G00000000978 | N/A | unclassified | 1.26 | 8.58E-03 |
|  | G00000019587 | Ptprn | protein tyrosine phosphatase, receptor type, N | 1.17 | 1.86E-02 |
|  | G00000011648 | Aqp1 | aquaporin 1 | 1.17 | 2.70E-02 |
|  | G00000018937 | Gstm2 | glutathione S-transferase mu 2 | 0.94 | 7.05E-05 |
|  | G00000003515 | Ephx1 | epoxide hydrolase 1 | 0.93 | 1.27E-02 |
|  | G00000009421 | Ivd | isovaleryl-CoA dehydrogenase | 0.93 | 4.42E-02 |
|  | G00000004009 | Xpnpep2 | X-prolyl aminopeptidase 2 | 0.92 | 9.78E-04 |
|  | G00000013949 | NADP | isocitrate dehydrogenase | 0.92 | 4.04E-02 |
|  | G00000010017 | Wee1 | WEE1 G2 checkpoint kinase | 0.79 | 3.19E-03 |
|  | G00000000645 | Reep3 | receptor accessory protein 3 | 0.76 | 4.59E-02 |
|  | G00000011747 | Tmem205 | transmembrane protein 205 | 0.71 | 2.70E-02 |
|  | G00000003038 | Sft2d2 | SFT2 domain containing 2 | 0.53 | 2.21E-02 |
| **PFC Down-regulated** | G00000009550 | Sqle | squalene epoxidase | -4.96 | 3.53E-14 |
|  | G00000016690 | Idi1 | isopentenyl-diphosphate delta isomerase 1 | -2.71 | 3.53E-14 |
|  | G00000020480 | Fads1 | fatty acid desaturase 1 | -2.61 | 5.31E-14 |
|  | G00000012819 | Gdnf | glial cell derived neurotrophic factor | -2.48 | 1.65E-03 |
|  | G00000007234 | Cyp51 | cytochrome P450, family 51 | -2.06 | 3.53E-14 |
|  | G00000006859 | Insig1 | insulin induced gene 1 | -1.96 | 8.06E-03 |
|  | G00000006280 | Pcsk9 | proprotein convertase subtilisin/kexin type 9 | -1.89 | 6.29E-05 |
|  | G00000016552 | Hmgcs1 | 3-hydroxy-3-methylglutaryl-CoA synthase 1 | -1.77 | 3.11E-07 |
|  | G00000036615 | RGD1560242 | similar to RIKEN cDNA 1700028P14 | -1.76 | 3.56E-02 |
|  | G00000032297 | Msmo1 | methylsterol monooxygenase 1 | -1.74 | 3.76E-14 |
|  | G00000011622 | Echdc1 | ethylmalonyl-CoA decarboxylase 1 | -1.65 | 3.32E-03 |
|  | G00000005871 | Il1rn | interleukin 1 receptor antagonist | -1.60 | 6.69E-03 |
|  | G00000043377 | Fdps | farnesyl diphosphate synthase | -1.39 | 3.81E-03 |
|  | G00000006787 | Dhcr24 | 24-dehydrocholesterol reductase | -1.36 | 9.88E-03 |
|  | G00000045636 | Fasn | fatty acid synthase | -1.34 | 9.14E-04 |
|  | G00000020704 | Tkfc | triokinase and FMN cyclase | -1.32 | 3.07E-02 |
|  | G00000016924 | Acly | ATP citrate lyase | -1.30 | 2.15E-04 |
|  | G00000016122 | Hmgcr | 3-hydroxy-3-methylglutaryl-CoA reductase | -1.25 | 1.43E-04 |
|  | G00000018755 | Acss2 | acyl-CoA synthetase short-chain family member 2 | -1.20 | 9.88E-04 |
|  | G00000032508 | Acot5 | acyl-CoA thioesterase 5 | -1.09 | 1.95E-02 |
|  | G00000002212 | 17-beta | hydroxysteroid | -1.08 | 2.36E-02 |
|  | G00000023348 | Tbc1d2 | TBC1 domain family, member 2 | -1.08 | 4.42E-02 |
|  | G00000000658 | Acacb | acetyl-CoA carboxylase beta | -1.02 | 2.59E-02 |
|  | G00000013387 | Tpcn2 | two pore segment channel 2 | -0.92 | 4.04E-02 |
|  | G00000046889 | Dbi | diazepam binding inhibitor, acyl-CoA binding protein | -0.89 | 3.39E-02 |
|  | G00000057814 | Nsdhl | NADP-dependent steroid dehydrogenase-like | -0.64 | 8.06E-04 |
|  | G00000004903 | Ebo | emopamil binding protein (sterol isomerase) | -0.64 | 2.60E-02 |
| **VAT Up-regulated** | G00000018237 | Gstp1 | glutathione S-transferase pi 1 | 1.89 | 1.65E-05 |
|  | G00000011250 | Inmt | indolethylamine N-methyltransferase | 1.78 | 3.42E-05 |
|  | G00000013484 | Gsta3 | glutathione S-transferase alpha-3 | 1.37 | 3.20E-03 |
|  | G00000058571 | N/A | unclassified | 1.14 | 3.76E-02 |
|  | G00000033206 | Entpd5 | ectonucleoside triphosphate diphosphohydrolase 5 | 1.10 | 2.57E-02 |
|  | G00000032745 | Slc17a3 | solute carrier family 17 member 3 | 1.06 | 5.08E-02 |
|  | G00000011573 | Csad | cysteine sulfinic acid decarboxylase | 1.01 | 2.49E-03 |
|  | G00000008755 | Acox1 | acyl-CoA oxidase 1 | 0.98 | 1.07E-02 |
|  | G00000047708 | Gstz1 | glutathione S-transferase zeta 1 | 0.92 | 2.08E-02 |
| **VAT Down-regulated** | G00000017672 | Akr1c14 | aldo-keto reductase family 1, member C14 | -3.43 | 2.66E-16 |
|  | G00000043451 | Spp1 | secreted phosphoprotein 1 | -3.14 | 3.20E-03 |
|  | G00000010047 | Ddit4l | DNA-damage-inducible transcript 4-like | -2.56 | 5.51E-03 |
|  | G00000013704 | Cps1 | carbamoyl-phosphate synthase 1 | -1.90 | 1.72E-03 |
|  | G00000010833 | Mthfd2 | methylenetetrahydrofolate dehydrogenase (NADP+ dependent) 2, methenyltetrahydrofolate cyclohydrolase | -1.79 | 3.24E-02 |
|  | G00000058739 | Snn | stannin | -1.56 | 5.51E-03 |
|  | G00000004626 | Slc34a2 | solute carrier family 34 member 2 | -1.54 | 4.45E-03 |
|  | G00000014453 | Anxa5 | annexin A5 | -1.52 | 3.81E-06 |
|  | G00000002579 | Parm1 | prostate androgen-regulated mucin-like protein 1 | -1.50 | 3.81E-03 |
|  | G00000003120 | Prelp | proline and arginine rich end leucine rich repeat protein | -1.48 | 3.63E-03 |
|  | G00000015550 | Ptgds | prostaglandin D2 synthase | -1.35 | 2.45E-02 |
|  | G00000018351 | Thap4 | THAP domain containing 4 | -1.22 | 3.81E-03 |
|  | G00000009019 | Slc6a6 | solute carrier family 6 member 6 | -1.19 | 5.08E-02 |
| **Liver Up-regulated** | G00000003144 | Gprc5c | G protein-coupled receptor, class C, group 5, member C | 2.13 | 4.43E-02 |
| **Liver Down-regulated** | G00000019422 | Egr1 | early growth response 1 | -2.06 | 1.04E-02 |

^1^All genes were analyzed using DESeq2 for differential analysis; no DEGs were detected in the kidney

^2^Abbreviations used: L2FC, log2 fold change; PFC, prefrontal cortex; VAT, visceral adipose tissue

^3^Benjamini-Hochberg adjusted P-values controlling for false discovery rate at 5%, where P< 0.05 was considered significant.

**Supplementary Table 4.** Kegg Pathway and Gene Ontology (GO) Analysis Representing Mapped Functional Pathways for Pooled Tissue-Specific DEGs^a,b^.

| **Tissue** | | | **ID** | **Term** | **#** | ***p*-value** | **Gene Symbols** |
| --- | --- | --- | --- | --- | --- | --- | --- |
| **PFC** | | GO:0006695 | cholesterol biosynthetic process | 8 | 3.90E-13 | *Hmgcr, Hmgcs1, sterol isomerase, Nsdhl, Dhcr24, Idi1, Fdps* |  |
|  | GO:0006633 | fatty acid biosynthetic process | 6 | 1.10E-07 | *Scd, Acsm3, Acacb, Fasn, Acly, Msmo1* |  |  |
|  | GO:0055114 | oxidation-reduction process | 12 | 8.70E-07 | *17-beta, Scd, Hmgcr, Fads1, Fasn, Sqle, Cyp2c22, Cyp51, Nsdhl, Dhcr24, Cyp4a1, Msmo1* |  |  |
|  | GO:0016126 | sterol biosynthetic process | 4 | 5.10E-06 | *Insig1, Sqle, sterol isomerase, Msmo1* |  |  |
|  | GO:0008610 | lipid biosynthetic process | 4 | 8.00E-06 | *Scd, Fasn, Acly, Acss2* |  |  |
|  | GO:0008203 | cholesterol metabolic process | 5 | 2.30E-05 | *Pcsk9, Insig1, Sqle, Nsdhl, Dhcr24* |  |  |
|  | GO:0008299 | isoprenoid biosynthetic process | 4 | 2.30E-05 | *Hmgcr, Hmgcs1, Idi1, Fdps* |  |  |
|  | GO:0006084 | acetyl-CoA metabolic process | 3 | 1.50E-04 | *Acacb, Fasn, Acly* |  |  |
|  | GO:0006641 | triglyceride metabolic process | 4 | 1.70E-04 | *Pcsk9, Scd, Insig1, Dbi* |  |  |
|  | GO:0014070 | response to organic cyclic compound | 6 | 7.80E-04 | *Hmgcs1, Fads1, Acacb, Ephx1, Il1rn, Gstm2* |  |  |
|  | GO:0006637 | acyl-CoA metabolic process | 3 | 2.50E-03 | *Acsm3, Acot5, Dbi* |  |  |
|  | GO:0019932 | second-messenger-mediated signaling | 2 | 8.00E-03 | *Gck, Ptprn* |  |  |
|  | GO:0043588 | skin development | 3 | 9.90E-03 | *Arrdc3, Dhcr24, Dbi* |  |  |
|  | KEGG | Metabolic pathways | 23 | 9.50E-10 | *Gck, Hmgcs1, Acsm3, Acacb, Fasn, NADP, sterol isomerase, Sqle, Hdc, Acly, Acot5, Nsdhl, Cyp51, Acss2, Dhcr24, Idi1, Cyp4a1, Tkfc, Fdps, Hmgcr, Cyp2c22, Ivd, Msmo1* |  |  |
|  | KEGG | Biosynthesis of antibiotics | 12 | 1.60E-09 | *Gck, Hmgcr, Hmgcs1, NADP, Sqle, Acly, Acss2, Cyp51, Nsdhl, Idi1, Msmo1, Fdps* |  |  |
|  | KEGG | Steroid biosynthesis | 6 | 2.10E-08 | *Sqle, sterol isomerase, Cyp51, Nsdhl, Dhcr24, Msmo1* |  |  |
|  | KEGG | Terpenoid backbone biosynthesis | 4 | 1.40E-04 | *Hmgcr, Hmgcs1, Idi1, Fdps* |  |  |
|  | KEGG | Propanoate metabolism | 3 | 7.00E-03 | *Echdc1, Acacb, Acss2* |  |  |
|  | KEGG | Biosynthesis of unsaturated fatty acids | 3 | 7.50E-03 | *Scd, Fads1, Acot5* |  |  |

| **VAT** | GO:0004364 | glutathione Transferase activity | 3 | 5.10E-04 | *GSTA3, GSTP1, GSTZ1* |
| --- | --- | --- | --- | --- | --- |
|  | GO:1903825 | organic acid transmembrane transport | 2 | 4.80E-03 | *Slc17a3, Slc6a6* |
|  | GO:0044341 | sodium-dependent phosphate transport | 2 | 7.70E-03 | *Slc17a3, Slc34a2* |
|  | GO:0006817 | phosphate ion transport | 2 | 9.70E-03 | *Slc17a3, Slc34a2* |
|  | GO:0005436 | sodium:phosphate symporter activity | 2 | 6.70E-03 | *Slc17a3, Slc34a2* |
|  | GO:0015321 | sodium-dependent phosphate transmembrane transporter activity | 2 | 7.70E-03 | *Slc17a3, Slc34a2* |
|  | KEGG | Metabolic pathways | 6 | 8.70E-03 | *Cps1, Mthfd2, Csad, Ptgds, Gstz1, Acox1* |

1. Pathway analyses were determined via DAVID
2. Abbreviations used: GO, gene ontology; KEGG, Kyto Encyclopedia of Genes and Genomes; PFC, prefrontal cortex, PFC; and VAT, visceral adipose tissue
3. *p*-values represent the probability of genes in the list annotated to a particular GO or KEGG pathway term. Threshold set at p < .001.

**Supplementary Table 5.** Differentially Expressed MicroRNAs between Sprague Dawley Rats Fed Dietary Whole Egg Compared to Casein Control.

| **Tissue** | **MicroRNA** | **Log2Fold** | **FDR non-adjusted P-value** |
| --- | --- | --- | --- |
| PFC Upregulated | rno-let-7e-5p | 1.339427 | 0.005181862 |
|  | rno-miR-30a-3p | 0.388272 | 0.029746435 |
|  | rno-miR-98-5p | 0.960443 | 0.035167986 |
| PFC Downregulated | rno-miR-10a-5p | -1.42826 | 0.025129776 |
|  | rno-miR-10b-5p | -1.35052 | 0.027749043 |
|  | rno-miR-29a-3p | -0.89674 | 0.03613351 |
|  | rno-miR-192-5p | -0.82033 | 0.057313629 |
| VAT Upregulated | rno-miR-221-3p | 0.8928 | 0.023787818 |
| VAT  Downregulated | rno-miR-140-3p | -1.01197 | 0.001986062 |
|  | rno-miR-125b-5p | -1.17509 | 0.007240392 |
|  | rno-miR-191a-5p | -0.7673 | 0.011564826 |
|  | rno-miR-10b-5p | -0.49055 | 0.033921745 |
| Liver Upregulated | rno-miR-30c-5p | 0.352427 | 0.027632486 |
|  | rno-miR-30d-5p | 0.32298 | 0.056044331 |
| Liver Downregulated | rno-miR-21-5p | -0.44345 | 0.003247421 |
|  | rno-miR-192-5p | -0.19144 | 0.021920638 |

^1^All miRNAs were analyzed using DESeq2 for differential analysis

^2^Abbreviations used: L2FC, log2 fold change; PFC, prefrontal cortex; and VAT, visceral adipose tissue.

^3^Benjamini-Hochberg adjusted P-values controlling for false discovery rate at 5%, where P< 0.05 was considered significant.
